# Success of community-based system dynamics in prevention interventions: A systematic review of the literature

**DOI:** 10.3389/fpubh.2023.1103834

**Published:** 2023-03-24

**Authors:** Tiana Felmingham, Kathryn Backholer, Elizabeth Hoban, Andrew D. Brown, Phoebe Nagorcka-Smith, Steven Allender

**Affiliations:** ^1^Global Centre for Preventive Health and Nutrition, Institute for Health Transformation, Deakin University, Geelong, VIC, Australia; ^2^School of Health and Social Development, Faculty of Health, Deakin University, Geelong, VIC, Australia

**Keywords:** systems thinking, community-based system dynamics, systematic review, success, evaluation, prevention

## Abstract

Systems thinking approaches are increasingly being used to help communities understand and develop responses to preventing complex health problems. Less is known about how success is characterized and what influences success in these approaches. We present a systematic review of how concepts of success are understood and evaluated in the peer reviewed literature of studies using systems thinking in community prevention. We searched five databases for peer-reviewed literature published between 2000 and 2022, with search terms related to systems thinking, prevention and community. Studies were included if they; reported using community-based systems thinking to prevent a public health problem; described the engagement and empowerment of community members to address a public health issue; and, were published in English. Thirty-four articles were identified from 10 countries. Twenty-one aimed to prevent a chronic disease (e.g., obesity) and 16 measured success using specific tools, 10 of which used semi-structured interviews or surveys. Measures of success included implementation processes, cultural appropriateness, the number or type of actions implemented, effectiveness of community action, and changes in individual thinking or mental models, population health outcomes, data collected, or systems level measures. Implementation factors influencing success included the capacity to engage participants, composition and experience of facilitators, strength of coordination teams, allocation of resources, adaptation to participant feedback, use of multiple systems approaches, workshop process providing time and methods to allow new insights, flexible delivery, and diversity of perspectives. Findings from each of the articles indicated that approaches increased a range of outcomes including community action, strategic thinking, future planning and evaluation, community buy-in, community voice, contribution and leadership, in addition to developing shared visions and goals and creating new, ongoing collaborations, among many others. Measures of success varied, suggesting more empirical reporting of proposed outcomes of system science in communities would be valuable. While the measurement of success in the use of systems thinking in community-based prevention efforts is limited, there are helpful examples we can look to for future measurement of success.

## 1. Introduction

The application of systems thinking to address complex community and social problems is gaining momentum (1, 2), particularly in community settings (3, 4). A range of methods have emerged within systems thinking that are used to help capture and engage with complexity inherent in many modern problems (5). According to Ison (p. 142) (5), systems thinking (or systemic thinking) in this instance is considered to be ‘*the understanding of a phenomenon within the content of a larger whole; to understand things systemically literally means to put them into context, to establish the nature of their relationships*.’

There are a wide range of systems thinking approaches, which are shaped by the various historical influences of systems practice across different disciplines (6). Common techniques to work with communities using system thinking include participatory system dynamics (PSD), group model building (GMB), soft systems methodology (7), critical systems heuristics (8) and community-based system dynamics (CBSD) (9, 10). While there is overlap between methods, there are also key distinctions, which generally span the level of involvement participants have in the process, the ownership participants have over the diagram developed and overall capacity built as a result of participant engagement (10). Most examples of systems thinking studies in the public health literature provide in-depth descriptions of the community’s understanding of a complex problem, but few provide insights on the effectiveness of the method, nor the implications of these methods for the success of attempts to address the problem overall. GMB stands out as one form of systems thinking with a greater amount of documented evaluation in the literature (3, 11, 12).

Long before systems thinking gained momentum in public health, community participation and engagement have been called for as a critical element in prevention efforts (13, 14). CBSD is an application of GMB that emphasizes participation and engagement alongside systems thinking (10). A key aspect of CBSD is engaging community or stakeholders in an agreed problem to gain shared insights and identify corresponding community-led action through the use of GMB (9, 10). This typically involves stakeholders in a series of workshops or consultations who create a diagram (in public health, often a causal loop diagram (CLD)) which helps visualize a complex problem from the community’s perspective. CBSD builds community capacity to recognize key feedback loops in a system’s structure that drive a system’s behavior, mobilizing action for systems change.

The concept of success can be contentious, and for the purpose of this review, success (or not) of an approach is considered in light of the authors conclusions within each article. While an approach is not considered completely ‘successful’ or ‘unsuccessful’, it is important to draw from past experiences that may have included components that helped facilitators get closer to their outcome, or those that may have created challenges.

While there are numerous descriptions of the use of CBSD to identify causal factors, interrelationships and actions to address a problem, much less literature describes the effectiveness or success of the approach. Within the literature that is available, findings are fragmented. No study has systematically searched the literature to examine success of CBSD across multiple studies, nor identified factors that influence success.

This systematic review assesses the current evidence describing success of CBSD and examines implementation factors that influence this success by asking the following research questions:

1. How is success in community-based system dynamics understood and measured in public health?
2. What implementation factors influence success of community-based system dynamics efforts in public health?

## 2. Methods

This review was registered with PROSPERO in January 2021 (CRD42021212817). Reporting of results was conducted in accordance with the Preferred Reporting Items for Systematic Reviews and Meta-Analyses (PRISMA) guidelines (15).

Our review focused on systems thinking approaches that specifically brought the community together to address the prevention of a public health or social problem, specifically using CBSD, or where a participatory method of visualization, modeling or causal diagram creation was applied to empower or mobilize a community in response to a complex problem. Definitions of CBSD by Hovmand (9) and descriptions by Király and Miskolczi (10) have been used to define the boundaries of this review.

### 2.1. Search strategy

The search was inclusive of empirical research published between January 2000 and October 2022. Both qualitative and quantitative study designs were included in our review. Only articles published in English were included.

Studies were searched using the MEDLINE complete, PsycInfo, CINAHL, Global Health and SocIndex databases. Search terms focused on three primary areas: community (population), systems thinking (intervention), prevention (outcome). Terms from the three areas were combined with the operator ‘AND’. Within the primary search areas, more specific search terms were combined with the operator “OR” (Table 1). The broad term of “systems thinking” was included as pilot literature searching identified there were few published studies that measure success of CBSD when using these terms alone.

**Table 1:** Search term concepts and variations.

Search term concept	Search term variation
Systems thinking	“system* science” OR “system dynamics” OR “system* thinking” OR “system* change*” OR “system* approach*” OR “system* initiative*” OR “system* theor*” OR “system* model*” OR “system* action*” OR (MH “systems theory”) OR “complex problem*” OR “complex adaptive system*” OR “complex system*” OR “group model building” OR “causal loop diagram*” OR “participatory system*” OR (MH “Nonlinear Dynamics”)
Prevention	“public health” OR “health promotion” OR “early intervention” OR “population health” OR “rural health” OR “urban health” OR prevent* OR “mental health” OR obesity OR alcohol OR “food system*” OR (MH “Public Health”) OR (MH “Preventive Medicine”) OR (MH “Primary Prevention”) OR (MH “Health Promotion”)
Community	communit* OR stakeholder*

The search strategy was adapted to the syntax requirements of each database. Reference lists of all included articles, and other relevant review articles identified, were additionally scanned for relevant studies.

All retrieved references were exported into the Endnote reference management software and transferred to Covidence, an online review platform, where duplicates were removed and articles were screened for inclusion.

### 2.2. Study selection

Use of CBSD terminology is sporadic. For the purpose of this paper, we will use the term CBSD when describing success, implementation and measures for all included articles in our review, even if the term has not been stated in the included article. This provides recognition of those articles using methods that encompass the principles of CBSD, as described in the following inclusion criteria.

Articles were included if they reported projects that; described collaboration or coalitions within specified communities; used, or described using an approach to systems thinking in the community setting (stated they were using CBSD, or alternatively, GMB, participatory systems or described building/using a qualitative CLD with the community); described engaging with stakeholders to apply systems thinking; focused on prevention of a public health issue; described a process that intended to empower individuals from a community (to take action, mobilize, or advocate); and had participation of community members across all stages of problem definition, diagram development, testing and transferring insights back into community. Studies were excluded if they did not consider community-level outcomes. An end point for the CBSD process was not defined in the criteria, as this varied and was highly dependent on what facilitators intended to see change as a result of using CBSD.

### 2.3. Screening process

The titles and abstracts of retrieved articles were independently screened by two members of the review team (TF, ADB or PNS) and discrepancies were resolved through discussion with a third reviewer (ADB or PNS). Before starting full text review, three authors involved in the screening process (TF, ADB, PNS) reviewed a subset of articles to ensure application of criteria were consistent. The remaining full text articles were reviewed by two independent authors (TF with ADB or PNS) with conflicts discussed and resolved between three authors (TF, ADB, PNS). Reasons for article exclusion at this stage were recorded. The most common reasons for exclusion were articles that were the wrong study type, wrong systems approach, or wrong health issue.

### 2.4. Data extraction and synthesis

Two reviewers (TF, ADB or PNS) independently extracted relevant data from 20% of articles (selected in alphabetical order, by first author) using a pre-specified and agreed upon data extraction template which included study title, intervention title (if specified), country or region of study/implementation, year of publication and implementation, author (s)/organization (s), study design, aim, nature of complex problem, lead implementation organization, collaborations, number and type of stakeholders involved in implementation (community members, professionals, others), details of the implementation process, method of data collection for success, and authors conclusions of the success of the process. Discrepancies were discussed within this sample to ensure consistency across the remainder of articles. Remaining data extraction was completed by one reviewer (TF).

**Figure 1:**
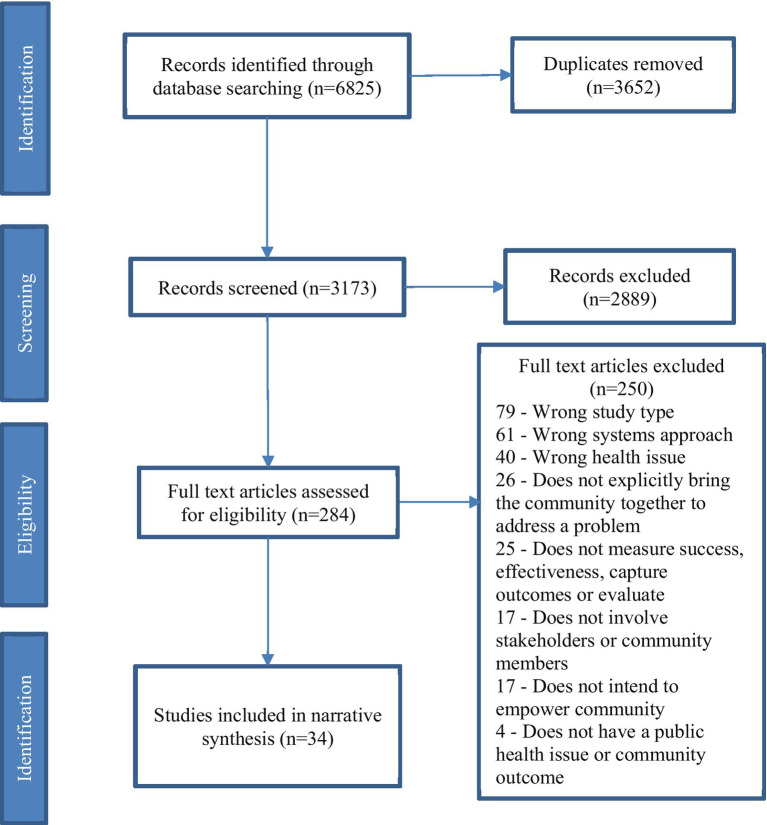
PRISMA flow chart.

The number of studies screened, assessed and included in the final review were recorded and reported using the PRISMA flowchart (Figure 1). As our study aimed to better understand the varying concepts of what constituted success in CBSD, a summary of the findings across the literature is presented (Supplementary Table 3). Results are grouped into studies that have used author observation to report on success and those that have used non-observation data collection methods.

### 2.5. Quality assessment

We did not undertake an assessment for the risk of bias as this was a systematic review with narrative synthesis intended to summarize the current state of the literature. We did however apply a standard approach to assessing the quality of studies returned by the review.

Study quality was assessed using the Critical Appraisal Skills Programme (CASP) Checklists (16). Quality appraisal was conducted by the primary investigator (TF) with 20% of articles cross-checked by one member of the review team to ensure consistency (ADB or PNS). Although no literature was excluded based on quality, discussion on quality of studies is included.

### 2.6. Analysis

A narrative synthesis, guided by Popay and Roberts (17), was used in this review due to the varying nature of study designs employed by those using CBSD. This involved; developing a preliminary synthesis of findings of included studies, exploring relationships in the data, and assessing the robustness of the synthesis (for example, quality assessments and quantity, of included articles, and minimizing bias by including multiple reviewers at each stage).

Descriptions provided by authors of each article were used to identify interventions (if they were named), regions where approaches occurred, the nature of the complex problem identified, and how systems thinking was used with each community.

Inductive thematic analysis (18) was used by one reviewer (TF) to explore themes within three subsets of the data extracted, specifically where; data related to concepts of success, identification and descriptions of implementation factors, and, overall findings (for example, how an implementation factor increased or decreased perceived success). The reviewer (TF) identified codes and categories as they emerged, and where codes or categories were identified as similar, themes emerged. The main, reoccurring or most important concepts were identified across all included studies by identifying those that occurred most often or were described by authors as having a critical influence on results. Results for this analysis are presented in sections 3.5 and 3.6.

## 3. Results

The search of all databases yielded 6,825 articles, before 3,652 duplicates were removed. A total of 3,173 titles and abstracts were screened, with 284 articles identified eligible for full-text review. Of these, 34 articles met the inclusion criteria and were included in this review (Supplementary Table 3).

### 3.1. Sample characteristics

The 34 articles represented 12 different interventions. Twenty articles did not specify an intervention name. Two articles related to The Whole of Systems Trial of Prevention Strategies for Childhood Obesity (WHOSTOPS) (19, 20), with three additional articles focused on subset interventions within WHOSTOPS [GenR8 Change (21)], Sustainable Eating Activity Change Portland [or SEA Change Portland (22) and Portland, a WHO STOPS pilot community (23)]. Two articles related to Healthy Families Waitākere (HFW) (24, 25), and the remaining seven articles described a single intervention (Campbelltown - Changing our Future (Change4Campbelltown) (26), Nourishing Hawke’s Bay: He wairua tō te kai (27), Prevention Impacts Simulation Model (PRISM) (28), Shape Up Under 5 (SUU5) (29), Derby: a City on the Move (DaCotM) (30), Urban Health in Latin America (“Salud Urbana en América Latina,” or SALURBAL) (31), and the Food & Fitness (F&F) Initiative (32).

The articles reviewed were of mixed quality and value, based on the Critical Appraisal Skills Programme (CASP) Checklists (16) (Table 2). One article was assessed as low value (33), 23 moderately valuable (21, 23, 24, 27, 28, 30, 34–50) and 10 were of high value (19, 20, 22, 25, 26, 29, 31, 32, 51, 52).

**Table 2:** Summary of quality assessment for included articles.

Articles	Qualitative studies (CASP Qualitative Studies Checklist)									
	1. Was there a clear statement of the aims of the research?	2. Is a qualitative methodology appropriate?	3. Was the research design appropriate to address the aims of the research?	4. Was the recruitment strategy appropriate to the aims of the research?	5. Was the data collected in a way that addressed the research issue?	6. Has the relationship between researcher and participants been adequately considered?	7. Have ethical issues been taken into consideration?	8. Was the data analysis sufficiently rigorous?	9. Is there a clear statement of findings?	Overall comments 10. How valuable is the research?
Allender et al., 2020	Yes	Yes	Yes	Cannot tell	Cannot tell	Yes	No	Yes	Yes	Excellent - very valuable
Bolton et al., 2022	Yes	Yes	Yes	Cannot tell	Yes	Cannot tell	Yes	Cannot tell	Yes	Moderately valuable
Brown et al., 2022	Yes	Yes	Yes	Cannot tell	Yes	Cannot tell	Yes	Cannot tell	Yes	Moderately valuable
Browne et al., 2021	Yes	Yes	Yes	Yes	Yes	Yes	Yes	Yes	Yes	Excellent - very valuable
Burke et al., 2014	Yes	Yes	Yes	Cannot tell	Cannot tell	Cannot tell	No	Cannot tell	Yes	Moderately valuable
Calancie et al., 2022	Yes	Yes	Yes	Yes	Yes	Cannot tell	No	Yes	Yes	Excellent—very valuable
Calancie et al., 2020	Yes	Yes	Yes	Yes	Yes	No	No	Yes	Yes	Excellent - very valuable
Cavill et al., 2020	Yes	Yes	Yes	No	Yes	Cannot tell	Cannot tell	Yes	Yes	Moderately valuable
Chavez-Ulgade et al., 2022	Yes	Yes	Yes	Yes	Yes	No	Yes	Yes	Yes	Moderately valuable
Deutsch et al., 2021	Yes	Yes	Yes	Cannot tell	Yes	Yes	No	Cannot tell	Yes	Moderately valuable
Egbuonye et al., 2022	Yes	Yes	Yes	Cannot tell	Yes	Cannot tell	No	No	Yes	Moderately valuable
Frerichs et al., 2018	Yes	Yes	Cannot tell	Cannot tell	Yes	Cannot tell	No	Yes	Yes	Moderately valuable
Frerichs et al., 2016	Yes	Yes	Yes	Yes	Cannot tell	Yes	No	Cannot tell	Yes	Moderately valuable
Gerritsen et al., 2020	Yes	Yes	Yes	Yes	Cannot tell	Cannot tell	Yes	No	Yes	Excellent - very valuable
Gerritsen et al., 2019	Yes	Yes	Yes	Cannot tell	Cannot tell	Cannot tell	Yes	No	Yes	Moderately valuable
Haroz et al., 2021	Yes	Yes	Yes	Cannot tell	Cannot tell	Cannot tell	No	Cannot tell	Yes	Moderately valuable
Jacobs et al., 2021	Yes	Yes	Cannot tell	No	Yes	Yes	Yes	No	Yes	Excellent - very valuable
Jenkins et al., 2020	Yes	Yes	Yes	Yes	Yes	No	Yes	Yes	Yes	Excellent - very valuable
Kumar et al., 2016	Yes	Yes	Yes	Cannot tell	Cannot tell	No	No	No	No	Not valuable for this review
Loyo et al., 2013	Yes	Yes	Yes	Yes	Yes	No	no	Cannot tell	Yes	Moderately valuable
Macmillan et al., 2016	Yes	Yes	Yes	yes	Yes	No	Yes	yes	Yes	Moderately valuable
Maitland et al., 2021	Yes	Yes	Yes	Yes	Yes	No	Yes	Yes	Yes	Excellent - very valuable
Marcal et al., 2021	Yes	Yes	Yes	Yes	Yes	No	No	Cannot tell	Yes	Moderately valuable
McKelvie-Sebileau et al., 2022	Yes	Yes	Yes	Yes	Cannot tell	Yes	Yes	Cannot tell	Yes	Moderately valuable
Morais et al., 2021	Yes	Yes	Yes	Yes	Yes	No	Yes	Yes	Yes	Excellent - very valuable
Mui et al., 2019	Yes	Yes	Yes	Cannot tell	Cannot tell	yes	Cannot tell	Cannot tell	Yes	Moderately valuable
Naumann et al., 2020	Yes	Yes	Yes	Yes	Cannot tell	No	Cannot tell	Cannot tell	Yes	Moderately valuable
Noubani et al., 2020	Yes	Yes	Yes	Yes	Cannot tell	Yes	Cannot tell	Cannot tell	Yes	Moderately valuable
Noubani et al., 2021	Yes	Yes	Yes	Yes	Cannot tell	Yes	Yes	Cannot tell	Yes	Moderately valuable
Sweirad et al., 2020	Yes	Yes	Yes	Yes	Cannot tell	No	No	Cannot tell	Yes	Moderately valuable
Trani et al., 2016	Yes	Yes	Yes	Cannot tell	Cannot tell	Yes	No	Cannot tell	Yes	Moderately valuable
Waqa et al., 2017	Yes	Yes	Yes	Yes	Cannot tell	Yes	Yes	Cannot tell	Yes	Moderately valuable
Zablith et al., 2021	Yes	Yes	Yes	Yes	Cannot tell	Yes	Cannot tell	Cannot tell	Yes	Moderately valuable
Zurcher et al., 2018	Yes	Yes	Yes	Yes	Yes	Yes	No	Yes	Yes	Excellent - very valuable
	Cluster control trial (CASP RCT Checklist)									
	1. Did the study address a clearly focused research question?	2. Was the assignment of participants to interventions randomized?	3. Were all participants who entered the study accounted for at its conclusion?	4. Were the participants ‘blind’ to intervention they were given? • Were the investigators ‘blind’ to the intervention they were giving to participants? • Were the people assessing/analyzing outcome/s ‘blinded’?	5. Were the study groups similar at the start of the randomized controlled trial?	6. Apart from the experimental intervention, did each study group receive the same level of care (that is, were they treated equally)?	7. Were the effects of intervention reported comprehensively?	8. Was the precision of the estimate of the intervention or treatment effect reported?	9. Do the benefits of the experimental intervention outweigh the harms and costs?	10. Can the results be applied to your local population/in your context? 11. Would the experimental intervention provide greater value to the people in your care than any of the existing interventions?
Jacobs et al., 2021	Yes	Yes	Yes	Cannot tell, No, No	Yes	Yes	Yes	Yes	Yes	Yes, Yes, Excellent, very valuable

### 3.2. Region or country

Articles described interventions that were implemented across 10 different countries or regions, with 26 articles describing interventions conducted within high income countries [13 from the United States (28, 29, 32, 35–37, 40, 44, 46–49, 52) with one article each related to PRISM and SUU5, with the remaining interventions not described, seven from Australia (19–23, 26, 51) with five articles connected to WHOSTOPS or subset interventions, one article related to the Change4Cambelltown intervention, and one intervention not described. There were three articles from New Zealand (24, 25, 27) two of which were related to the HFW intervention and one connected to Nourishing Hawke’s Bay: He wairua tō te kai. Three articles were from the United Kingdom (30, 34, 45)], one of which was connected to the DaCotM intervention, with the remaining two interventions not described. Four articles described interventions in upper middle income countries, in Lebanon (38, 39, 43) and Thailand (50), with all focused on refugee communities (it is worth noting that all interventions in Lebanon occurred in areas with high disadvantage, including those areas with newly arrived Syrian refugees, and the intervention in Thailand was related to those living in refugee camps), none of which identified a name for their interventions. One article described an intervention in Fiji (42), a middle income country, while two articles described interventions in lower middle income countries [one from India (33), one from the Latin American region (31)]. Of these, all were unnamed interventions, with the exception of the SALURBAL intervention in Latin America. One article described an unnamed intervention in Afghanistan (41), one of the world’s lowest income countries (53).

### 3.3. Complex problems

Of the articles included, 10 described interventions focused on childhood obesity (19–22, 27, 29, 40, 45, 48, 52), two on childhood fruit and vegetable intake (24, 25), and one on childhood overweight and obesity (26). The articles reported on findings from seven interventions: SUU5 (29), WHOSTOPS (19, 20), two additional interventions connected to WHOSTOPS (GenR8 Change (21) SEA Change Portland (22)), HFW (24, 25), Change4Campbelltown (26) and Nourishing Hawke’s Bay: He wairua tō te kai (27). Four articles that focused on childhood obesity described interventions that were unnamed (40, 45, 48, 52).

In addition, eight articles reported findings on CBSD approaches that focused on some aspect of chronic disease. This includes the following topics: chronic disease as an outcome (44), burden of chronic disease (28), non-communicable disease (43)), and changing environments to encourage physical activity and healthy eating (physical inactivity (30), availability of healthy foods in low income communities (36), use of evidence in food related policy making (42), water and sugar sweetened beverage consumption (23) and healthy eating and active living (32)) for the population overall.

Three articles reported on interventions that focused on mental health of refugee and local communities (38, 39, 41), all of which occurred in Lebanon and Afghanistan. Three articles focused on equity (racial inequity (47), health equity (31), and inequities experienced by Indigenous women in relation to intimate partner violence and alcohol misuse (46)). Two articles focused on housing (housing, energy and wellbeing (34) and family homeless shelter use (35)).

Other complex problems reported include; community violence (49), road traffic safety and pedestrian deaths (37) and sustained adoption of cleaner cooking technologies (33). One article did not describe the focus of interventions specifically, as its aim was to explore the use of CBSD in Indigenous communities in Australia across various interventions (51).

### 3.4. Use of systems thinking with the community

Twenty-six of the 34 articles used CBSD in the community in addition to testing or refining the systems method used. Four articles used CBSD in the community alone, without intention to test or refine the method, nor use it as part of evaluation. Three articles described using systems thinking to test and refine the method and the remaining article used systems thinking as an evaluation technique. Twelve articles described using CBSD as part of a wider intervention, with 20 articles describing stand alone interventions. In two articles it was unclear whether the CBSD approach was stand alone or part of a wider intervention.

Eighteen articles did not describe the composition of the facilitation team, eight articles identified that facilitation team members included a mix of academics and community leaders or professionals, seven stated facilitation teams comprised of academic researchers, and one identified a consultancy led facilitation.

Authors used terminology other than CBSD to describe their method of community or stakeholder engagement and qualitative model development (Table 3). Thirteen articles (13 interventions) explicitly describe using CBSD (20, 23, 27, 31, 33, 35, 36, 38, 40, 41, 46, 50, 51). The remaining 21 articles (20 interventions) use other descriptions to explain the methods they use. Five articles (five interventions) describe using GMB with the community to build a causal loop diagram (22, 24, 37, 42, 43), and three articles (three interventions) describe using GMB with the community (25, 29, 49). Three articles (three interventions) describe building a causal loop diagram with the community (26, 30, 48), two articles (two interventions) describe using system dynamics (SD) with the community (28, 44) and two articles (two interventions) describe using participatory GMB (21, 39).

**Table 3:** Summary of articles and interventions.

Author/s	Title of intervention	Nature of the complex problem	Context for use of systems thinking	Implementation process reported by authors	Participants/stakeholders involved in the intervention	Method of data collection for success of systems thinking approach	Quality check (CASP) How valuable is the research?
Allender et al. (20)	The Whole of Systems Trial of Prevention Strategies for Childhood Obesity (WHOSTOPS)	Childhood obesity	Using systems in community and testing the method	Methods inspired by CBSD and GMB to build a CLD	Leaders including health services, school principals, local government, councilors, retail leaders, business leaders, and key community figures.	Author reflection	Excellent—very valuable
Bolton et al. (21)	GenR8 Change, part of WHOSTOPS	Childhood obesity	Using systems in community and testing the method	GMB’s plus additional community workshops	Community leaders and members who designed and implemented interventions on behalf of children. Participants varied across workshops. Data session—15 community leaders, 5 working group members (local shire council representing 15% of the overall group), health and medical services (35%), PCP (15%), state government (5%), local and regional sporting organizations (10%), employment agency (5%), and the education sector (15%). GMB 1- not stated. GMB2 - not stated. GMB3–171 participants	Causal loop diagram with highlighted areas of action in GenR8 Change 12 months post-GMB3.	Moderately valuable
Brown et al. (23)	Portland, a WHO STOPS pilot community	Water and sugar sweetened beverage consumption	Using systems in community and testing the method	CBSD to build a SD model	11 key stakeholders from Portland with an interest or role in consumption of SSBs or water and included representatives from the Primary Care Partnership, local government, health service, sporting clubs, the local water authority, and community members	Author reflection	Moderately valuable
Browne et al. (51)	Various	Not described - various interventions	Testing or refining systems as a method	CBSD and GMB	Not described - various interventions	Qualitative semi-structured telephone/video conference interviews (individual and small group interviews)	Excellent—very valuable
Burke et al. (28)	Prevention Impacts Simulation Model (PRISM)	Burden of chronic diseases	Testing or refining systems as a method	System dynamics model to inform community-level policy decisions.	Members of both the local public health department and community members participated in building the model	Case studies - comparison of systems methods using RE-AIM	Moderately valuable
Calancie et al. (52)	Not described	Obesity	Using systems in community and testing the method	Stakeholder-Driven Community Diffusion (SDCD) -informed intervention that uses GMB	12 key stakeholders selected from the Early Ages Healthy Stages (EAHS) Coalition, EAHS leaders identified 10 Committee members, with input from the research team on sector representation. The 2 remaining positions were chosen by coalition-wide nomination. The Committee represented 8 sectors: nutrition assistance programs, early education, center-based childcare, home-based childcare, public health department, community-based organization, private business, and philanthropy.	Online surveys and interviews to assess Committee member perspective shifts, and a follow-up survey to identify actions taken by the EAHS following the SDCD-informed intervention with the Committee. Surveys were administered during months 5 and 9 of Committee meetings. Interviews with Committee members at baseline and at the conclusion of the study. The same interview questions were asked at both points. Follow-up action survey - Fourteen months after the conclusion of Committee meetings, the research team distributed another online survey to all members. This survey was different than the one used to assess shifts in perspectives.	Excellent—very valuable
Calancie et al. (29)	Shape Up Under 5	Childhood obesity	Using systems in community and testing the method	Community-based process for using GMB	The SUU5 Committee was composed of 16 professionals from early childhood education and care (n = 5), parks and recreation (n = 2), the local health department (n = 2), health care (n = 3), food assistance programs (n = 1), and the public schools (n = 3)	Exit survey at the end of each meeting (measuring knowledge, engagement, and trust). In addition, measuring perspective shifts using two formats: an online survey at 3 time points (1 year, 18 months, and 2 years from the beginning of the project) and semi structured interviews at 2 time points (1 and 2 years after baseline)	Excellent - very valuable
Cavill et al. (30)	‘Derby: a City on the Move (DaCotM)’	Physical inactivity	Using systems in community and testing the method	Systems mapping with communities to build CLDs	The DaCotM consortium - local government organizations, registered charities and further and higher education providers	Semi-structured interviews approximately 6 months after systems maps had been drafted and discussed. Meeting notes and written comments from the mapping sessions (approximately 12–15 attendees per session) were used to corroborate the findings from the interviews where possible.	Moderately valuable
Chavez-Ugalde et al. (45)	Not described	Obesity	Using systems in community and testing the method	GMB adapted online	GMB’s - 11 adolescents, 10 from Bristol Young People’s Advisory Group (YPAG) and 1 from Avon Scouts. Additional workshop - Public health practitioners and policymakers	Brief anonymous online feedback survey	Moderately valuable
Deutsch et al. (46)	Not described	Intimate partner violence (IPV) and alcohol misuse (AM), with a focus on inequities experienced by Northern Plains Indigenous women.	Using systems in community and testing the method	A case study from a CBSD project	Northern Plains Indigenous Women. Stakeholder partners include both those with personal and professional experience, and public, non-profit and grassroots organizations. Participants receiving services from Group 1: a faith-based re-entry programs for women who were previously incarcerated; Group 2: a substance use treatment program for pregnant women and mothers; and Group 3: a domestic violence shelter. One modeling session held within each organization. Group 1 – five women, Group 2–20 women, Group 3 - four women. Did not collect identifying information from participants for anonymity. However, learned during the sessions that majority of participants in each group self-identified as Indigenous (although this was never asked explicitly by the session facilitators).	Author reflection	Moderately valuable
Egbuonye et al. (47)	Not described	Equity	Using systems in community and testing the method	A participatory action approach of dynamic system mapping and systemic strategy design	76 stakeholders, including representatives from health care, mental health, education, economic development, faith, human services, and government.	Author reflection	Moderately valuable
Frerichs et al. (48)	Not described	Childhood obesity	Testing or refining systems as a method	Produce visual diagrams that highlighted system structures. Youth produced two types of systems diagrams: (a) graphs over time and (b) CLDs	Twenty-one adolescent African American youths	Survey at baseline and immediately after each of the four sessions. Semi structured interviews with youth postintervention with both high and low levels of participation.	Moderately valuable
Frerichs et al. (49)	Not described	Community violence	Using systems in community and testing the method	Develop, adapt, and apply GMB methods	6-member core planning team plus 27 individuals: 11 from academic research settings, 16 community partners representing law enforcement, schools, housing, grassroots community organizations, religious institutions, and prior gang-involved youth. Participants were diverse in gender and race.	Adaptations to GMB on advice from diverse community members, in addition to post-satisfaction survey and qualitative feedback	Moderately valuable
Gerritsen et al. (25)	Healthy Families Waitākere (HFW)	Fruit and vegetable intake among children	Using systems in community and testing the method	A GMB process that engaged members of a diverse urban community	17 participants (14 of whom attended all three workshops)	Informal feedback or meetings at three times points - during and immediately after implementation of workshops (informal feedback), three months after the final workshop (partnership meeting held), and 12 months after workshops (met with staff from HFW to discuss what had happened in the interim with the purpose of evaluating the benefits and impact of the GMB process)	Excellent - very valuable
Gerritsen et al. (24)	Healthy Families Waitākere (HFW)	Fruit and vegetable intake among children	Using systems in community and testing the method	GMB to create a CLD	Local retailers, health promoters, schools and the wider community, with a minimum of two from each of these sectors. Secondary school students were included if they were over 16 years of age. A total of 17 community members participated in the three workshops. All main ethnic groups (Māori, Pacific, Asian and NZ European) were represented, with over half of participants identifying as Māori or Pacific	Author reflection	Moderately valuable
Haroz et al. (50)	Not described	Suicide prevention	Using systems in the community	CBSD	Two refugee camps on the border of Thailand and Myanmar. Towns of Mae Sot which is close to Mae La camp and Umphang - the western border of Thailand. Local stakeholders from organizations working with displaced populations in Thailand, along with experts on systems modeling, suicide prevention, health systems, humanitarian contexts, and global mental health. Summaries from each workshop were presented in three languages (Karen, Burmese and English). The first workshop was held in Mae Sot, and included 21 participants representing organizations working with refugee, internally displaced person (IDP), and migrant populations. The second workshop was held in Umphang and included eight participants representing organizations working with refugee populations. A third workshop was held, which included nine participants with expertise in systems approaches, suicide prevention, global mental health, and humanitarian contexts. Many of the workshop participants were from the displaced and migrant communities in the area (representing Karen and Burman ethnicities). A final workshop was held in Mae Sot, and consisted of 14 stakeholders from organizations working with refugee populations.	Author reflection	Moderately valuable
Jacobs et al. (19)	WHOSTOPS	Childhood obesity	Using systems in community and testing the method	A systems-based CBI approach, to develop a causal loop diagram	Leaders in the five intervention communities	Three monitoring waves (2015, 2017 and 2019). School participation rates, Height and weight data, weight-related behaviours and HRQoL of Grade 4 and 6 students were collected by self-report questionnaire. The Index of Community Socio-Educational Advantage (ICSEA) scores for each school were used as an indicator of SEP. The average of height and weight measures was used to calculate body mass index z-scores (BMI-z). Data on gender and age were collected for Year 2 students. Year 4 and 6 students were guided through questionnaires - gender, date of birth, language usually spoken at home, Aboriginal and/ or Torres Strait Islander background, residential postcode, and country of birth. The Core Indicators and Measures of Youth Health – Physical Activity & Sedentary Behaviour Module questionnaire was used to assess PA and sedentary behaviour and active transport. The Simple Dietary Questionnaire, which is based on the Australian Dietary Guidelines, was used to assess dietary behaviours. Health related quality of life was assessed using the 23-item Paediatric Quality of Life Inventory 4.0 (PedsQL)	Excellent - very valuable
Jenkins et al. (22)	Sustainable Eating Activity Change Portland (SEA Change Portland), part of WHOSTOPS	Childhood obesity	Using systems in community and testing the method	GMB to develop CLD’s with community participation	Not described	Semi-structured interviews and a focus group	Excellent - very valuable
Kumar et al. (33)	Not described	Sustained adoption of cleaner cooking technologies	Using GMB as an evaluation technique	A CBSD modeling approach	Number of participants not identified. GMB sessions were primarily conducted with women.	Author reflection	Not valuable for this review
Loyo et al. (44)	Not described	Chronic disease	Using systems in community and testing the method	A system dynamics model shared with stakeholders in the context of a multistakeholder “action lab”	56 participants attended the action lab, representing a range of public health, health care, nonprofit, advocacy groups, businesses, and schools. There was comprehensive representation across intervention areas except for air quality, which was represented indirectly by people working in the area of tobacco or asthma. Each participant also belonged to at least one community-based coalition, and many were key leaders.	Informal feedback – on completion participants were asked to rate their perceived levels of commitment, influence, and confidence in making the changes they had identified as most necessary.	Moderately valuable
Macmillan et al. (34)	Not described	Housing, energy and wellbeing	Using systems in community and testing the method	Participatory system dynamics modelling. A combination of primary and secondary data was used to develop a CLD and included individual semi-structured interviews with participants using cognitive mapping.	Over 50 stakeholders, representing 37 organizations. These included six national government departments; five representatives from local government; 14 non-government organizations; a group of six minority-ethnicity housing leaders (community roots group); five industry organizations; and eight academic institutions. Some stakeholders represented more than one sector.	Author reflection	Moderately valuable
Maitland et al. (26)	Campbelltown - Changing our Future (Change4Campbelltown)	Childhood overweight and obesity	Using systems in community and testing the method	A stakeholder-informed CLD.	Not described	Action register, stakeholder engagement database, GANTT chart for timeline and grant reporting requirements, actions represented on a CLD, communication log	Excellent - very valuable
Marçal et al. (35)	Not described	Family homeless shelter use	Using systems in community and testing the method	A CBSD study, that utilized GMB and key informant interviews to develop a causal feedback theory of factors	37 homeless clients with children. Participants were overwhelmingly female (91%) and Black (87%), and two-thirds were first-time shelter clients (65%). The mean age was 39.6 (SD ¼ 13.0) years. Families on average included 2.5 children (SD ¼ 1.8), family size ranged from 1 to 5 children. Staff participants were all female and primarily Black (83%). Agency employment tenures ranged from five to 24 years. Interviews were conducted with an executive director, a shelter manager, and a case manager who offered perspectives on client experiences of shelter stays and their own experiencing as providers.	Author reflection	Moderately valuable
McKelvie-Sebileau et al. (27)	Nourishing Hawke’s Bay: He wairua t ¯o te kai	Childhood obesity	Using systems in the community	CBSD	Hawke’s Bay region – Key stakeholders - District Health Board, Iwi (tribal group), school principals and Ministry of Education. Over the three workshops, 19 rangatahi (youth) from five regional high schools, and 26 community stakeholders participated. The high schools comprised of two low decile (1–3) schools (low community advantage) and three mid-decile (4–7) schools (mid community advantage). Community stakeholders represented 24 organizations including - District Health Board, Ministry of Education, kaupapa M¯aori health providers and trusts, Iwi, Heart Foundation, Eastern Institute of Technology School of Health Science, Hawke’s Bay Community Fitness Centre Trust, Sport Hawke’s Bay, food rescue charity, local food production business representatives and a supermarket owner, as well as teachers from Early Learning Services and low advantage primary schools. Of the 26 adults participating, approximately half were of M¯aori ethnicity. No demographic information was taken and individuals to ensure privacy and confidentiality.	Author reflection	Moderately valuable
Morais et al. (31)	Urban Health in Latin America (“Salud Urbana en América Latina,” or SALURBAL)	Health equity in Latin America	Using systems in community and testing the method	CBSD workshops	24 experts (São Paulo workshop) in food systems and transportation sectors working primarily in Brazil, with regional, national, and international influence, including “elected and administrative policy-makers, members of civil society (e.g., nonprofits), and academics.”	Semi-structured interviews, 12 months after the São Paulo workshop	Excellent - very valuable
Mui et al. (36)	Not described	Availability of healthy foods in low income urban communities	Using systems in community and testing the method	CBSD to elicit perspectives from diverse stakeholders	18 participants, representing a diverse group comprising: 3 chain and local storeowners, 8 community residents, 3 representatives from city government agencies, and 4 representatives from local non-profit organizations.	Author reflection	Moderately valuable
Naumann et al. (37)	Not described	Road traffic safety - pedestrian deaths	Using systems in community and testing the method	A systems mapping technique (ie, CLDs) within a GMB context to identify a wide range of ‘mental models’.	41 stakeholders, participants represented: pedestrian and bicycle advocacy, law enforcement, automobile industry, academia/research, health department, medical professions, local government, city planning, transit department, department of transportation and social services.	Author reflection	Moderately valuable
Noubani et al. (38)	Not described	Mental health	Using systems in community and testing the method	CBSD, through GMB workshops or semi- structured interviews.	89 participants from both contexts and communities. A diverse gender-and age-balanced group of both Syrian refugees and Lebanese host community members. General community members (adults aged over 18) and caretakers of people affected by MHPSS issues (e.g., parents of children aged 10–18). Lebanese community - 2 GMB workshops (Beirut - 9 females, 7 males; Beqaa - 9 females, 3 males), 18 semi-structured interviews (Beirut - 5 females, 4 males; Beqaa - 6 females, 3 males). Syrian refugees - 2 GMB workshops (Beirut - 10 females, 6 males; Beqaa - 2 females, 7 males) 18 semi-structured interviews (Beirut - 5 females, 4 males; Beqaa - 5 females, 4 males).	Author reflection	Moderately valuable
Noubani et al. (39)	Not described	Mental health	Using systems in the community	Participatory GMB workshops	36 health care providers active in mental health service provision (at least 1 year) from Beirut and Beqaa regions, 15 semi structured interviews conducted with psychologists, nurses, social workers and general practitioners across genders, 21 participants participated in two GMB workshops	Author reflection	Moderately valuable
Swierad et al. (40)	Not described	Childhood obesity	Using systems in community and testing the method	CBSD	16 Chinese American adults. All participants were aged between 20 and 60 years, and 43.8% (7/16) were male. Six participants were born overseas. Participants represented a variety of occupations including nurses, school guidance counselors, restaurant owners, community health workers, and housewives.	Author reflection	Moderately valuable
Trani et al. (41)	Not described	Mental health	Using systems in community and testing the method	A CBSD-informed GMB workshop	Initial sessions - three male and three female community based rehabilitation workers from the Mazar-e-Sharif region and four male CBR workers from Jalalabad. Four participants in the follow-up sessions were from Mazar-e-Sharif, Taloqan, Ghazni and Jalalabad, four regional program offices of the partner NGO.	Author reflection	Moderately valuable
Waqa et al. (42)	Not described	Evidence use in food-related policymaking	Using systems in community and testing the method	GMB and a system dynamics approach	18 participants from the MoHMS (n = 9) and the MOA (n = 9). The majority of participants (72%) were senior managers (such as National Advisors, Directors and Principal level officers) directly involved in policymaking, 28% were middle with potential to share evidence that influences the policymaking process. The majority (72%) were male.	Author reflection	Moderately valuable
Zablith et al. (43)	Not described	Non-communicable diseases	Using systems in the community	Semi-structured interviews followed by GMB workshops.	67 participants. 30 semi-structured interviews: 10 health care providers (physicians, pharmacists, nurses, PHCC managers, 5 male) in the Beqaa, 10 Lebanese (3 men, age range overall 23–60) and 10 Syrian refugee (3 men, age range overall 30–60) community members. All community participants suffered from a chronic condition or self-identified as being at risk of NCD development. First GMB - 10 health care providers (one physician, two pharmacists, six nurses, one PHCC manager); participants had between 3 and 15 years’ experience of working in the Beqaa. Second and third GMB 12 Lebanese community members (41% male, age range 20–50), 15 Syrian refugees (13% male, age range 24–55). All community participants self-identified as having an NCD or a risk factor.	Author reflection	Moderately valuable
Zurcher et al. (32)	Food & Fitness (F&F) Initiative	Healthy eating and active living	Using systems in community and testing the method	Five key frameworks and a systems mapping process with community and initiative leaders - Core Theory of Success (CTS), Creative Tension Model (CTM), Hierarchy of Choices (HOC), Levels of Perspective (LoP), Ladder of Inference (LoI), causal loop diagramming	24 grassroots and institutional members of the six final grantee communities, 10 TA providers, and four WKKF staff participated in structured conversations at the conference. An additional 12 individuals for interviews, ranging from grassroots community members to local evaluators and project directors.	Structured conversations and in-depth phone interviews.	Excellent - very helpful

Other methods described include; participatory system dynamics modeling (SDM) with the community (34), systems frameworks including CLD’s (32), Stakeholder-Driven Community Diffusion (SDCD) using GMB (52), GMB online with community (45), a participatory action approach of dynamic system mapping and systemic strategy design (47), and a systems-based community-based intervention (CBI) approach, to build a CLD (19).

### 3.5. Measuring success

All articles reported on more than one outcome (Table 4), identifying 14 themes, which included measuring contribution, engagement and collaborative experience, cultural appropriateness, the implementation process, ownership, trust and relationships, implementation of action, ongoing community engagement and community voice, and unintended consequences. Changes in individual thinking, insights, ideas or mental models, organizational commitment, the system or social norms, data collection, data sources or measurement of change, individual health outcomes, prevention practice, and support for action were also measured.

**Table 4:** Measures of success by author for 16 articles that measure success.

Author	Bolton et al., 2022	Browne et al., 2021	Burke et al., 2014	Calancie et al., 2022	Calancie et al., 2020	Cavill et al., 2020	Chavez-Ugalde et al., 2022	Frerichs et al., 2018	Frerichs et al., 2016	Gerritsen et al., 2020	Jacobs et al., 2021	Jenkins et al., 2020	Loyo et al., 2013	Maitland et al., 2021	Morais et al., 2021	Zurcher et al., 2018
Measuring success by…																
Measuring contribution, engagement and collaborative experience	✓	✓	✓	✓	✓	✓	✓	✓	✓	✓		✓	✓	✓	✓	✓
Measuring cultural appropriateness		✓							✓							
Measuring the implementation process		✓				✓	✓	✓	✓	✓				✓	✓	✓
Changes in individual thinking /insights/ideas/mental models		✓	✓	✓	✓	✓	✓	✓	✓	✓		✓	✓		✓	
Measuring ownership, trust and relationships	✓	✓		✓	✓		✓		✓	✓		✓		✓	✓	✓
Support to take action	✓		✓		✓			✓		✓			✓			
Changes in organizational commitment	✓				✓											
Changes in the system/social norms	✓					✓					✓					✓
Changes in data collection, data sources and measurement of change	✓					✓								✓		✓
Changes in health outcomes						✓					✓					
Measuring implementation of action	✓			✓						✓			✓	✓		✓
Measuring ongoing community engagement and community voice	✓									✓		✓		✓		
Changes in prevention practice												✓		✓	✓	✓
Measuring unintended consequences																✓

Of the 34 articles, 18 describe the success of their intervention through subjective author observation and reflection (20, 23, 24, 27, 33–43, 46, 47, 50), that is, where authors describe the success of the intervention, in the absence of additional data collection. The remaining 16 articles use a range of other methods to measure success or effectiveness of the intervention.

The 18 articles that describe their success through author observation and reflection include four named interventions across four articles, WHOSTOPS (20) HFW (24), Portland, a WHOSTOPS pilot community (23) and Nourishing Hawke’s Bay: He wairua tō te kai (27). Fourteen articles did not identify a named intervention.

The remaining 16 articles measured success or effectiveness of their approach using semi-structured interviews at different timepoints (22, 29–31, 48, 51, 52), surveys or questionnaires at different timepoints (19, 29, 45, 48, 49, 52), informal qualitative feedback at different timepoints (25, 44, 49), project documentation (for example meeting minutes) (26, 30), action tracking (action register or on CLD) (21, 26), health measures and population health or education datasets (19), comparative systems thinking case studies (28), stakeholder engagement database (26) and structured conversations and in-depth interviews (32) (Table 5).

**Table 5:** Evaluation method by author for 16 articles that measure success.

Author		Bolton et al., 2022	Browne et al., 2021	Burke et al., 2014	Calancie et al., 2022	Calancie et al., 2020	Cavill et al., 2020	Chavez-Ugalde et al., 2022	Frerichs et al., 2018	Frerichs et al., 2016	Gerritsen et al., 2020	Jacobs et al., 2021	Jenkins et al., 2020	Loyo et al., 2013	Maitland et al., 2021	Morais et al., 2021	Zurcher et al., 2018
Data collection method																	
Qualitative semi-structured interviews (individual or small group)	Before implementation (baseline)				✓												
	During implementation												✓				
	At delayed timepoint/s post implementation		✓		✓	✓	✓		✓							✓	
Surveys or questionnaires	Before implementation (baseline)				✓				✓			✓					
	During implementation				✓	✓			✓			✓					
	Immediately post implementation				✓			✓		✓		✓					
	At delayed timepoint/s post implementation				✓	✓											
Informal qualitative feedback	During implementation to adapt scripts and workshops									✓	✓						
	Immediately post implementation										✓						
	At delayed timepoint/s post implementation										✓			✓			
Individual health measurements and demographics												✓					
Population health and education datasets												✓					
Structured conversations and in-depth interviews																	✓
Tracking actions (action register or on CLD)		✓													✓		
Stakeholder engagement database															✓		
Project documentation							✓										
Comparative systems thinking case studies				✓													

### 3.6. What influences success

There were numerous implementation factors influencing findings from across the studies. Nineteen themes emerged during the analysis of implementation factors. Of the articles describing success through author observation, the following themes describing implementation factors influenced success: the development of CLD’s; the overall process (including workshops or interviews, CBSD, GMB and other methods); the time allocated to the process (for example, categories included the length of a workshop or the length of the process overall) alongside the timing of different parts of the process (for example, categories included the time taken between workshops, or the time allowed for participants and facilitators to adapt to momentum); the participants who were engaged, methods of engagement and ongoing commitment; and the composition, skills and experience of the facilitation team (Table 6). While one article (20) touched on these issues, it included a focus on building capacity of the use of CBSD in prevention more broadly. As such, this paper identified additional factors as important to outcomes of the approach: supporting a strong process, including utilization of existing structures and ensuring strong collaborative relationships between practice and academia; and using a capacity building approach.

**Table 6:** Key implementation factors and how they affected findings in 16 studies that measured success.

Implementation factors that were identified as important	Findings from 16 articles that measure success	
The overall workshop/diagram approach	•Increased shared learning and story telling •Uncovered complexities, interconnections, changed thinking and created new insights •Increased cultural appropriateness •Increased focus on collaboration and shared vision •Allowed flexible delivery •Increased community voice •Increased knowledge, engagement and an awareness of who else should be ‘in the room’	•Changed prevention practice •Changed data collection and measurement approaches •Format and concepts were a challenge •Increased local capacity •Increased community-led action •Further development of methods •Community CLD was not used to its potential •Built trust and ownership •Increased shared learning and story telling
CLD’s and other diagrams	•Uncovered complexities, interconnections, changed thinking and created new insights •Increased community-led action •Increased focus on collaboration and shared vision •Test local scenarios and change decisions •Participation in evaluation was positive	•Changed data collection and measurement approaches •Further development of methods •Increased knowledge, engagement and an awareness of who else should be ‘in the room’ •Increased local capacity
Participants and engagement	•Built trust and ownership •Increased knowledge, engagement and an awareness of who else should be ‘in the room’ •Changed prevention practice •Increased focus on collaboration and shared vision	•Increased local capacity •Increased community-led action •Limited interest, capacity, time and miscommunication of the approachUncovered complexities, interconnections, changed thinking and created new insights
Facilitation team	•Increased local capacity •Increased community voice	•Built trust and ownership
Flexible delivery	•Allowed flexible delivery	•Increased cultural appropriateness
Coordination group or meetings	•Created new, ongoing networks •Increased knowledge, engagement and an awareness of who else should be ‘in the room’ •Increased community-led action	•Uncovered complexities, interconnections, changed thinking and created new insights •Changed prevention practice •Competing priorities limited engagement •Built trust and ownership
Implementing collective action	•Uncovered complexities, interconnections, changed thinking and created new insights •Increased community-led action •Increased knowledge, engagement and an awareness of who else should be ‘in the room’ •Changed data collection and measurement approaches	•Created systems change •Format and concepts were a challenge •Participation in evaluation methods and/or measuring change was difficult •Allowed flexible delivery •Built trust and ownership •Strong foundation for change
Time and timing	•Increased knowledge, engagement and an awareness of who else should be ‘in the room’	•Limited the opportunity to discuss ideas
Obtaining participant feedback	•Participation in evaluation methods and/or measuring change was difficult	
Leveraging workshop/diagram development outputs	•Increased community voice	•Increased community-led action
Use of systems thinking methods	•Community CLD was not used to its potential •Created systems change •Changed data collection and measurement approaches •Changed prevention practice	•Increased knowledge, engagement and an awareness of who else should be ‘in the room’ •Increased focus on collaboration and shared vision
Relationships and collaboration	•Strong foundation for change •Increased knowledge, engagement and an awareness of who else should be ‘in the room’ •Reduced unintended consequences	•Uncovered complexities, interconnections, changed thinking and created new insights •Increased focus on collaboration and shared vision •Increased community-led action
Multiple approaches combined	•Changed prevention practice •Created systems change	•Increased focus on collaboration and shared vision •Further development of methods
Flexible project evolution	•Allowed flexible delivery •Built trust and ownership	•Strong foundation for change
Resources	•Increased knowledge, engagement and an awareness of who else should be ‘in the room’	•Strong foundation for change
Data collection	•Participation in evaluation methods and/or measuring change was difficult •Increased knowledge, engagement and an awareness of who else should be ‘in the room’	•Increased focus on collaboration and shared vision Built trust and ownership •Changed prevention practice •Changed data collection and measurement approaches
A shared vision	•Increased community-led action	•Increased knowledge, engagement and an awareness of who else should be ‘in the room’
Aligning new and existing efforts	•Increased knowledge, engagement and an awareness of who else should be ‘in the room’	
Diverse perspectives	•Uncovered complexities, interconnections, changed thinking and created new insights	

Descriptions of the effects of implementation factors varied across articles, for example, where time and timing influenced success, one article (48) stated participants:

*“found the diagramming activities acceptable, but indicated they needed more time because they were only beginning to understand the concepts when the session ended.”*

Another article (26) identified:

*“Actions operated on differing timescales, for many there was some delay between the initial planning and the implementation and following there was often adaptation of the action.”*

While a third article (32) stated:

*“Every group and every individual interviewed emphasized that systems change in communities takes more time than people are accustomed to.”*

Additional implementation factors that influenced success in the 16 articles that used non-observation methods were; the role of coordination teams with the opportunity to shape the approach; the ability for a group to come together to implement collective action; providing opportunities for participant feedback on the process; leveraging workshop outputs; the use of systems thinking methods; the strength and quality of relationships and collaboration; the opportunity to combine multiple approaches simultaneously; and a flexible delivery model (accommodating for differences in language, number and timing of workshops, literacy, numeracy, computer literacy or confidence using technology) (Table 6).

Findings from the 16 articles are presented alongside implementation factors in Table 6 with 23 themes identified. Findings included increased community action, increases in strategic thinking, future planning and evaluation, increasing community buy-in and community voice, developing shared visions and goals and creating new, ongoing collaborations. Findings also included building momentum, increasing community contribution and leadership, acknowledging the time required to develop new partnerships and collective thinking, increased understanding of feedback and how it contributes to understanding problems and corresponding action, among many others.

## 4. Discussion

### 4.1. Summary of main findings

Measures and concepts of success varied across the articles reviewed, often comprising subjective observations and reflections of study authors, or resulting from semi-structured interviews with key stakeholders. Typical measures of success included community action, collaboration, changes in mental models, or cultural appropriateness. It is difficult to determine specific effects of each implementation factor theme as this was reported to vary across studies (for example, where one had a reported effect in one study, the same factor may have had the opposite effect in another study), which was also reportedly influenced by the characteristics of participants and the context of each approach. We found variation in methods to achieve the same outcome and variation in outcomes sought using the same method describing an emerging field trialing multiple alternate approaches.

Our review builds on reviews by Carey (1), Rusoja (2), and Wilkinson (54) who aimed to investigate the use of systems science or systems thinking in public health or health generally. All three reviews identified various systems methods and terms, with modeling, specifically causal loop diagrams, featuring as one of the most common systems methods used in health. Wilkinson found that although there were many calls to use systems thinking methods, there were few published examples. Our review shows growth in the application of systems thinking, specifically CBSD, and while example evaluations are limited, they are also beginning to grow. Calls from Carey (1) Rouwette (55) and Scott (11) highlight the important stretch beyond model creation as an outcome, to draw attention to research that examines the quality and effectiveness of systems science as a method, with Rouwette specifically calling for examination of successful and unsuccessful efforts (in GMB).

Our review supports findings in a review by Bagnall, Radley (56) that identifies barriers and enablers to implementation of whole systems approaches (WSA’s), noting that leadership, engagement, paying attention to partnerships and building trust (and allowing the time required to do so), governance and shared values, and developing collaborative teams all influenced success. Building on Bagnall’s review, Jayasinghe, Soward (57) found that WSA’s need to include as many domains of capacity building as possible. Domains identified the importance of leadership, in all its forms, alongside partnerships, community engagement and mobilization of resources, all of which were echoed as important implementation factors for success of CBSD approaches in our review. Cilenti, Issel (3) and Littlejohns, Hill (58) have also conducted helpful reviews that explore how system dynamics and CLD’s can be used by communities to realize community action. They found, as in our review, there are times when success may be defined in ways other than community action. This should be considered in future systematic reviews of CBSD and other community-based systems thinking methods.

A recent review (59) developed a framework for measuring success of participatory modeling in systems approaches, and though the study was not focused on community intervention alone, clear similarities were observed between the findings of the current study. Specifically they consider four categories (feasibility, value, change and action, and sustainability) to guide evaluation design of participatory modeling programs. Further parallels exist outside the use of system science, and the findings of this review are supported by other reviews of community capacity and readiness, for example Nagorcka-Smith, Bolton (60) reviewed 26 studies and found shared decision making, resourcing, leadership and facilitation were critical to successful implementation of community initiatives that involved community coalitions. This is echoed by Brush, Mentz (61) who identified the strength of relationships, characteristics and composition of the group influenced success.

### 4.2. Strengths and limitations

A limitation of this review was the wide variability of terms within systems thinking and the ability to identify those explicitly intending to empower and mobilize a community (as identified in descriptions by Hovmand (9) and Király and Miskolczi (10)). For this reason, the search terms ‘community’ and ‘stakeholder’ were applied to narrow the field of articles down to those most relevant in the community setting, although this may prove overly simplistic without the inclusion of alternatives (for example, using the term ‘participant’). In addition, systems thinking does not include other participatory modeling techniques such as ‘concept mapping’ or ‘group concept mapping’ (62) which were excluded from this review. Other systems methods, such as social network analysis (63), and agent-based modeling (64), were also excluded.

This review did not explore the grey literature as it was anticipated there would be far less empirical measurement of outcomes meaning several examples of CBSD may have been excluded from this review. The review is also limited to articles in the English language. Of 34 papers, few set out to actively evaluate their approach and only 16 had data extracted that could provide insights into implementation factors and measures of success. While some articles collected data at multiple timepoints, others did not, which proved challenging during analysis.

Strengths of this review include the systematic review of all published literature, with all titles, abstracts and full text screened by at least two authors. Analysis was conducted in line with the recommended process for narrative synthesis by Popay and Roberts (17), and in line with PRISMA guidelines (15). An additional strength was the breadth of terms used, which ensured inclusion of findings of other studies that may not have explicitly identified CBSD in their methodology. Adaptation of methods and scripts to apply CBSD is critical to the success of the practice (9). This review captures variations in the approach and compares these against adaptations to measure success based on the direction of the intervention.

### 4.3. Implications for policy and practice

Despite limited evidence for measurement of success CBSD, our systematic review shows that success is influenced by early design decisions, for example, identifying key stakeholders, composition of the facilitation team/s, timing of workshops, and perceived influence of the process by community members. This indicates it is not only crucial to consider and plan for measurement of success early in the application of CBSD, but that this planning will directly influence implementation, potentially influencing longer term outcomes for the community involved in the approach.

While this review focuses on the use of CBSD in prevention, findings may also be helpful to inform design, implementation and evaluation of other methods that aim to empower community to address complex problems, specifically measures of success, data collection methods, and implementation factors that influence success.

Summarizing the findings from this review for practice, we have developed the following considerations, with each informing the next:

- Consider why you are using CBSD with the community, and be clear on its purpose. What are you aiming to do? What is your end point?
- Identify what change to measure. What will change as a result of you using this approach? Is it community action, individual thinking, change in policy, approach to planning, increased collaboration, increased engagement, or another measure?
- Identify the most appropriate data collection tools. What data collection tools will you use to measure the change you are aiming for? If using surveys, are validated or tested surveys or questionnaires available? If the tool used is novel, is it described in enough detail that it can be replicated by others?
- Consider strategies that will support empowerment of the community after diagram development is complete. How will you care for participants and their contribution after diagram development is complete?

### 4.4. Future research

In the absence of extensive literature that measures success in CBSD, future research may benefit in looking to studies from other disciplines that have measured effectiveness of group model building (for example, organizational management and organizational change (65)) to draw on other existing and tested frameworks and tools. Examples include Rouwette’s previously developed questionnaire designed to measure communication quality, consensus and commitment to conclusions for those studies aiming to measure insight or collaboration (66), or Fokkinga’s mental model survey (67) for those aiming to measure changes in individual thinking. Measurement frameworks and tools required will vary and will be shaped by the original purpose and setting of the work.

Future attempts to measure success of CBSD should ensure insights are collected from community members and stakeholders at multiple timepoints, including at delayed timepoints after workshops or a CLD has been first developed. This will help determine how community members have applied new insights from the process, and help identify changes in actions, policy or planning at the local level. Evaluation frameworks applied in other participatory modeling approaches may also be helpful. Our review echoes calls from Lee, Hickie (59), emphasizing that evaluation is planned and budgeted for, extending beyond the development of the diagram or CLD. In the case of CBSD, it is important studies plan to capture impacts that stretch beyond modeling and explore impacts within the community.

## 5. Conclusion

Greater emphasis on measurement of success of future CBSD approaches is required. The use of CBSD and other community-based systems approaches in prevention is growing rapidly. Continuing to synthesize and apply evidence from this research as it emerges will be critical to improve population health. Research teams, alongside the communities they are working with, must articulate what they are seeking to change by using CBSD. Defining success in this way will help identify what will be measured, how it may be measured (including identifying appropriate data collection methods and tools) and will provide a clearer understanding of success.

There are helpful attempts to measure success and effectiveness of CBSD approaches in the published literature. These examples show the importance of design, facilitation strengths and ongoing community engagement as key factors in implementation.

## Data availability statement

The original contributions presented in the study are included in the article/Supplementary material, further inquiries can be directed to the corresponding author.

## Author contributions

TF completed the original search, contributed to article screening, full text assessment, data extraction, analysis, preparing initial and ongoing drafts of manuscript, and editing final manuscript. KB contributed to research design, draft manuscripts, and editing final manuscript. EH contributed to draft manuscripts and editing final manuscript. AB and PN-S contributed to article screening and assessment of full texts, draft manuscripts and final manuscript. SA contributed to final research design, provided ongoing supervision, draft manuscripts and editing final manuscript. All authors contributed to the article and approved the submitted version.

## Funding

TF holds a research role funded by VicHealth (Victorian Health Promotion Foundation). KB is supported by Heart Foundation Future Leader Fellowship (102047) from the National Heart Foundation of Australia. SA receives/has received funding from the Australian National Health and Medical Research Council (GNTs PPRG 2015440 Promoting CHANGE, GNT1151572 RESPOND, GNT1114118 WHO STOPS, GNT2013563,GNT10458360, GNT2011209, and GNT2002234 PRECIS) the Australian Medical Research Future Fund (DELIVER), VicHealth, Western Alliance, Barwon Health, the European Union Horizon 2020 H2020-SFS-2016-2017 (Co-Create), Novo Nordisk, UK Medical Research Council, Cancer Council Victoria, Deakin University, Melbourne Lord Mayor’s Charitable Foundation, The Australian Prevention Partnership Centre.

## Conflict of interest

The authors declare that the research was conducted in the absence of any commercial or financial relationships that could be construed as a potential conflict of interest.

## Publisher’s note

All claims expressed in this article are solely those of the authors and do not necessarily represent those of their affiliated organizations, or those of the publisher, the editors and the reviewers. Any product that may be evaluated in this article, or claim that may be made by its manufacturer, is not guaranteed or endorsed by the publisher.

## Abbreviations

CBSD: Community based system dynamics
GMB: Group model building
CLD: Causal loop diagram
