# Supplementary figures and images for: Success of community-based system dynamics in prevention interventions: A systematic review of the literature
Source: Front Public Health. 2023 Mar 24;11:1103834. doi: 10.3389/fpubh.2023.1103834 (PMC10080052; doi:10.3389/fpubh.2023.1103834)

## Supplementary Material

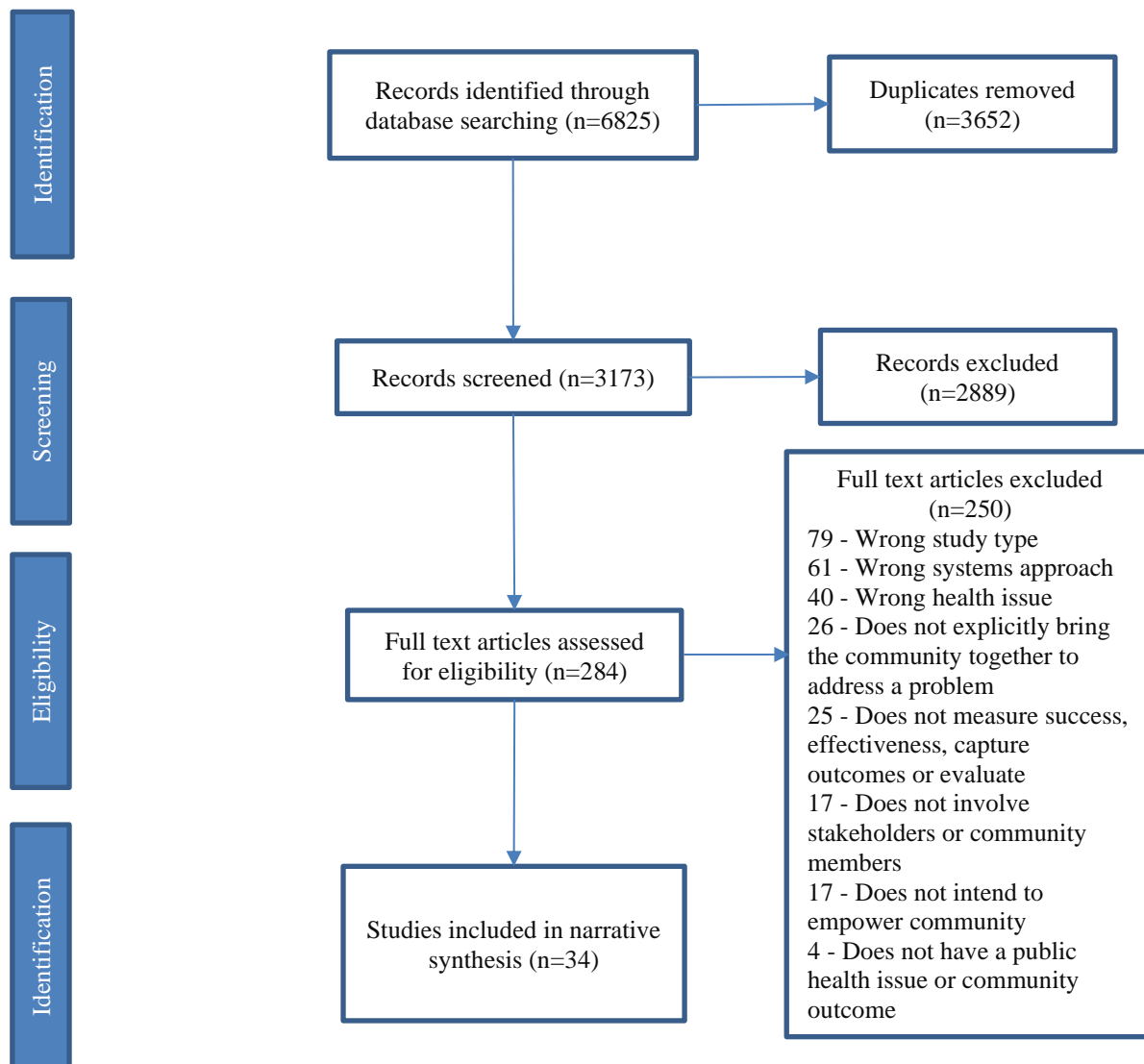

**Supplementary Figure 1. PRISMA Flowchart**

Supplement: Supplementary file 1 [file Image_1.pdf]
