# Supplementary material for: Success of community-based system dynamics in prevention interventions: A systematic review of the literature
Source: Front Public Health. 2023 Mar 24;11:1103834. doi: 10.3389/fpubh.2023.1103834 (PMC10080052; doi:10.3389/fpubh.2023.1103834)
Supplement: Supplementary file 2 [file Table_3.docx]

Table 3. Summary of articles and interventions

| **Author/s** | **Title of intervention** | **Nature of the complex problem** | **Context for use of systems thinking** | **Implementation process reported by authors** | **Participants/stakeholders involved in the intervention** | **Method of data collection for success of systems thinking approach** | **Quality check (CASP) How valuable is the research?** |
| --- | --- | --- | --- | --- | --- | --- | --- |
| Allender et al. (20) | The Whole of Systems Trial of Prevention Strategies for Childhood Obesity (WHOSTOPS) | Childhood obesity | Using systems in community and testing the method | Methods inspired by CBSD and GMB to build a CLD | Leaders including health services, school principals, local government, councillors, retail leaders, business leaders, and key community figures. | Author reflection | Excellent - very valuable |
| Bolton et al. (21) | GenR8 Change, part of WHOSTOPS | Childhood obesity | Using systems in community and testing the method | GMB's plus additional community workshops | Community leaders and members who designed and implemented interventions on behalf of children. Participants varied across workshops. Data session - 15 community leaders, 5 working group members (local shire council (representing 15% of the overall group), health and medical services (35%), PCP (15%), state government (5%), local and regional sporting organisations (10%), employment agency (5%), and the education sector (15%). GMB 1- not stated. GMB2 - not stated. GMB3 - 171 participants | Causal loop diagram with highlighted areas of action in GenR8 Change 12 months post-GMB3. | Moderately valuable |
| Brown et al. (23) | Portland, a WHO STOPS pilot community | Water and sugar sweetened beverage consumption | Using systems in community and testing the method | CBSD to build a SD model | 11 key stakeholders from Portland with an interest or role in consumption of SSBs or water and included representatives from the Primary Care Partnership, local government, health service, sporting clubs, the local water authority, and community members | Author reflection | Moderately valuable |
| Browne et al. (51) | Various | Not described - various interventions | Testing or refining systems as a method | CBSD and GMB | Not described - various interventions | Qualitative semi-structured telephone/video conference interviews (individual and small group interviews) | Excellent - very valuable |
| Burke et al. (28) | Prevention Impacts Simulation Model (PRISM) | Burden of chronic diseases | Testing or refining systems as a method | System dynamics model to inform community-level policy decisions. | Members of both the local public health department and community members participated in building the model | Case studies - comparison of systems methods using RE-AIM | Moderately valuable |
| Calancie et al. (52) | Not described | Obesity | Using systems in community and testing the method | Stakeholder-Driven Community Diffusion (SDCD) -informed intervention that uses GMB | 12 key stakeholders selected from the Early Ages Healthy Stages (EAHS) Coalition, EAHS leaders identified 10 Committee members, with input from the research team on sector representation. The 2 remaining positions were chosen by coalition-wide nomination. The Committee represented 8 sectors: nutrition assistance programs, early education, center-based childcare, home-based childcare, public health department, community-based organization, private business, and philanthropy. | Online surveys and interviews to assess Committee member perspective shifts, and a follow-up survey to identify actions taken by the EAHS following the SDCD-informed intervention with the Committee. Surveys were administered during months 5 and 9 of Committee meetings. Interviews with Committee members at baseline and at the conclusion of the study. The same interview questions were asked at both points. Follow-up action survey - Fourteen months after the conclusion of Committee meetings, the research team distributed another online survey to all members. This survey was different than the one used to assess shifts in perspectives. | Excellent - very valuable |
| Calancie et al. (29) | Shape Up Under 5 | Childhood obesity | Using systems in community and testing the method | Community-based process for using GMB | The SUU5 Committee was composed of 16 professionals from early childhood education and care (n = 5), parks and recreation (n = 2), the local health department (n = 2), health care (n = 3), food assistance programs (n = 1), and the public schools (n = 3) | Exit survey at the end of each meeting (measuring knowledge, engagement, and trust). In addition, measuring perspective shifts using two formats: an online survey at 3 time points (1 year, 18 months, and 2 years from the beginning of the project) and semi structured interviews at 2 time points (1 and 2 years after baseline) | Excellent - very valuable |
| Cavill et al. (30) | ‘Derby: a City on the Move (DaCotM)’ | Physical inactivity | Using systems in community and testing the method | Systems mapping with communities to build CLDs | The DaCotM consortium - local government organisations, registered charities and further and higher education providers | Semi-structured interviews approximately six months after systems maps had been drafted and discussed. Meeting notes and written comments from the mapping sessions (approximately 12-15 attendees per session) were used to corroborate the findings from the interviews where possible. | Moderately valuable |
| Chavez‑Ugalde et al. (45) | Not described | Obesity | Using systems in community and testing the method | GMB adapted online | GMB’s - 11 adolescents, 10 from Bristol Young People’s Advisory Group (YPAG) and 1 from Avon Scouts. Additional workshop - Public health practitioners and policymakers | Brief anonymous online feedback survey | Moderately valuable |
| Deutsch et al. (46) | Not described | Intimate partner violence (IPV) and alcohol misuse (AM), with a focus on inequities experienced by Northern Plains Indigenous women. | Using systems in community and testing the method | A case study from a CBSD project | Northern Plains Indigenous Women. Stakeholder partners include both those with personal and professional experience, and public, non-profit and grassroots organizations. Participants receiving services from Group 1: a faith-based re-entry programme for women who were previously incarcerated; Group 2: a substance use treatment programme for pregnant women and mothers; and Group 3: a domestic violence shelter. One modelling session held within each organization. Group 1 – five women, Group 2 - 20 women, Group 3 - four women. Did not collect identifying information from participants for anonymity. However, learned during the sessions that majority of participants in each group self-identified as Indigenous (although this was never asked explicitly by the session facilitators). | Author reflection | Moderately valuable |
| Egbuonye et al. (47) | Not described | Equity | Using systems in community and testing the method | A participatory action approach of dynamic system mapping and systemic strategy design | 76 stakeholders, including representatives from health care, mental health, education, economic development, faith, human services, and government. | Author reflection | Moderately valuable |
| Frerichs et al. (48) | Not described | Childhood obesity | Testing or refining systems as a method | Produce visual diagrams that highlighted system structures. Youth produced two types of systems diagrams: (a) graphs over time and (b) CLDs | Twenty-one adolescent African American youths | Survey at baseline and immediately after each of the four sessions. Semi structured interviews with youth postintervention with both high and low levels of participation. | Moderately valuable |
| Frerichs et al. (49) | Not described | Community violence | Using systems in community and testing the method | Develop, adapt, and apply GMB methods | 6-member core planning team plus 27 individuals: 11 from academic research settings, 16 community partners representing law enforcement, schools, housing, grassroots community organizations, religious institutions, and prior gang-involved youth. Participants were diverse in gender and race. | Adaptations to GMB on advice from diverse community members, in addition to post-satisfaction survey and qualitative feedback | Moderately valuable |
| Gerritsen et al. (25) | Healthy Families Waitākere (HFW) | Fruit and vegetable intake among children | Using systems in community and testing the method | A GMB process that engaged members of a diverse urban community | 17 participants (14 of whom attended all three workshops) | Informal feedback or meetings at three times points - during and immediately after implementation of workshops (informal feedback), three months after the final workshop (partnership meeting held), and 12 months after workshops (met with staff from HFW to discuss what had happened in the interim with the purpose of evaluating the benefits and impact of the GMB process) | Excellent - very valuable |
| Gerritsen et al. (24) | Healthy Families Waitākere (HFW) | Fruit and vegetable intake among children | Using systems in community and testing the method | GMB to create a CLD | Local retailers, health promoters, schools and the wider community, with a minimum of two from each of these sectors. Secondary school students were included if they were over 16 years of age. A total of 17 community members participated in the three workshops. All main ethnic groups (Māori, Pacific, Asian and NZ European) were represented, with over half of participants identifying as Māori or Pacific | Author reflection | Moderately valuable |
| Haroz et al. (50) | Not described | Suicide prevention | Using systems in the community | CBSD | Two refugee camps on the border of Thailand and Myanmar. Towns of Mae Sot which is close to Mae La camp and Umphang - the western border of Thailand. Local stakeholders from organizations working with displaced populations in Thailand, along with experts on systems modeling, suicide prevention, health systems, humanitarian contexts, and global mental health. Summaries from each workshop were presented in three languages (Karen, Burmese and English). The first workshop was held in Mae Sot, and included 21 participants representing organizations working with refugee, internally displaced person (IDP), and migrant populations. The second workshop was held in Umphang and included eight participants representing organizations working with refugee populations. A third workshop was held, which included nine participants with expertise in systems approaches, suicide prevention, global mental health, and humanitarian contexts. Many of the workshop participants were from the displaced and migrant communities in the area (representing Karen and Burman ethnicities). A final workshop was held in Mae Sot, and consisted of 14 stakeholders from organizations working with refugee populations. | Author reflection | Moderately valuable |
| Jacobs et al. (19) | WHOSTOPS | Childhood obesity | Using systems in community and testing the method | A systems-based CBI approach, to develop a causal loop diagram | Leaders in the five intervention communities | Three monitoring waves (2015, 2017 and 2019). School participation rates, Height and weight data, weight-related behaviours and HRQoL of Grade 4 and 6 students were collected by self-report questionnaire. The Index of Community Socio-Educational Advantage (ICSEA) scores for each school were used as an indicator of SEP. The average of height and weight measures was used to calculate body mass index z-scores (BMI-z). Data on gender and age were collected for Year 2 students. Year 4 and 6 students were guided through questionnaires - gender, date of birth, language usually spoken at home, Aboriginal and/ or Torres Strait Islander background, residential postcode, and country of birth. The Core Indicators and Measures of Youth Health – Physical Activity & Sedentary Behaviour Module questionnaire was used to assess PA and sedentary behaviour and active transport. The Simple Dietary Questionnaire, which is based on the Australian Dietary Guidelines, was used to assess dietary behaviours. Health related quality of life was assessed using the 23-item Paediatric Quality of Life Inventory 4.0 (PedsQL) | Excellent - very valuable |
| Jenkins et al. (22) | Sustainable Eating Activity Change Portland (SEA Change Portland), part of WHOSTOPS | Childhood obesity | Using systems in community and testing the method | GMB to develop CLD's with community participation | Not described | Semi-structured interviews and a focus group | Excellent - very valuable |
| Kumar et al. (33) | Not described | Sustained adoption of cleaner cooking technologies | Using GMB as an evaluation technique | A CBSD modeling approach | Number of participants not identified. GMB sessions were primarily conducted with women. | Author reflection | Not valuable for this review |
| Loyo et al. (44) | Not described | Chronic disease | Using systems in community and testing the method | A system dynamics model shared with stakeholders in the context of a multistakeholder “action lab” | 56 participants attended the action lab, representing a range of public health, health care, nonprofit, advocacy groups, businesses, and schools. There was comprehensive representation across intervention areas except for air quality, which was represented indirectly by people working in the area of tobacco or asthma. Each participant also belonged to at least one community-based coalition, and many were key leaders. | Informal feedback – on completion participants were asked to rate their perceived levels of commitment, influence, and confidence in making the changes they had identified as most necessary. | Moderately valuable |
| Macmillan et al. (34) | Not described | Housing, energy and wellbeing | Using systems in community and testing the method | Participatory system dynamics modelling. A combination of primary and secondary data was used to develop a CLD and included individual semi-structured interviews with participants using cognitive mapping. | Over 50 stakeholders, representing 37 organisations. These included six national government departments; five representatives from local government; 14 non-government organisations; a group of six minority-ethnicity housing leaders (community roots group); five industry organisations; and eight academic institutions. Some stakeholders represented more than one sector. | Author reflection | Moderately valuable |
| Maitland et al.(26) | Campbelltown - Changing our Future (Change4Campbelltown) | Childhood overweight and obesity | Using systems in community and testing the method | A stakeholder-informed CLD. | Not described | Action register, stakeholder engagement database, GANTT chart for timeline and grant reporting requirements, actions represented on a CLD, communication log | Excellent - very valuable |
| Marçal et al. (35) | Not described | Family homeless shelter use | Using systems in community and testing the method | A CBSD study, that utilized GMB and key informant interviews to develop a causal feedback theory of factors | 37 homeless clients with children. Participants were overwhelmingly female (91%) and Black (87%), and two-thirds were first-time shelter clients (65%). The mean age was 39.6 (SD ¼ 13.0) years. Families on average included 2.5 children (SD ¼ 1.8), family size ranged from 1 to 5 children. Staff participants were all female and primarily Black (83%). Agency employment tenures ranged from five to 24 years. Interviews were conducted with an executive director, a shelter manager, and a case manager who offered perspectives on client experiences of shelter stays and their own experiencing as providers. | Author reflection | Moderately valuable |
| McKelvie-Sebileau et al. (27) | Nourishing Hawke’s Bay: He wairua t ¯o te kai | Childhood obesity | Using systems in the community | CBSD | Hawke’s Bay region – Key stakeholders - District Health Board, Iwi (tribal group), school principals and Ministry of Education. Over the three workshops, 19 rangatahi (youth) from five regional high schools, and 26 community stakeholders participated. The high schools comprised of two low decile (1–3) schools (low community advantage) and three mid-decile (4–7) schools (mid community advantage). Community stakeholders represented 24 organizations including - District Health Board, Ministry of Education, kaupapa M¯aori health providers and trusts, Iwi, Heart Foundation, Eastern Institute of Technology School of Health Science, Hawke’s Bay Community Fitness Centre Trust, Sport Hawke’s Bay, food rescue charity, local food production business representatives and a supermarket owner, as well as teachers from Early Learning Services and low advantage primary schools. Of the 26 adults participating, approximately half were of M¯aori ethnicity. No demographic information was taken and individuals to ensure privacy and confidentiality. | Author reflection | Moderately valuable |
| Morais et al. (31) | Urban Health in Latin America (“Salud Urbana en América Latina”, or SALURBAL) | Health equity in Latin America | Using systems in community and testing the method | CBSD workshops | 24 experts (São Paulo workshop) in food systems and transportation sectors working primarily in Brazil, with regional, national, and international influence, including “elected and administrative policy-makers, members of civil society (e.g., nonprofits), and academics”. | Semi-structured interviews, 12 months after the São Paulo workshop | Excellent - very valuable |
| Mui et al. (36) | Not described | Availability of healthy foods in low income urban communities | Using systems in community and testing the method | CBSD to elicit perspectives from diverse stakeholders | 18 participants, representing a diverse group comprising: 3 chain and local storeowners, 8 community residents, 3 representatives from city government agencies, and 4 representatives from local non-profit organizations. | Author reflection | Moderately valuable |
| Naumann et al. (37) | Not described | Road traffic safety - pedestrian deaths | Using systems in community and testing the method | A systems mapping technique (ie, CLDs) within a GMB context to identify a wide range of ‘mental models’. | 41 stakeholders, participants represented: pedestrian and bicycle advocacy, law enforcement, automobile industry, academia/research, health department, medical professions, local government, city planning, transit department, department of transportation and social services. | Author reflection | Moderately valuable |
| Noubani et al. (38) | Not described | Mental health | Using systems in community and testing the method | CBSD, through GMB workshops or semi- structured interviews. | 89 participants from both contexts and communities. A diverse gender- and age-balanced group of both Syrian refugees and Lebanese host community members. General community members (adults aged over 18) and caretakers of people affected by MHPSS issues (e.g. parents of children aged 10–18). Lebanese community - 2 GMB workshops (Beirut - 9 females, 7 males; Beqaa - 9 females, 3 males), 18 semi-structured interviews (Beirut - 5 females, 4 males; Beqaa - 6 females, 3 males). Syrian refugees - 2 GMB workshops (Beirut - 10 females, 6 males; Beqaa - 2 females, 7 males) 18 semi-structured interviews (Beirut - 5 females, 4 males; Beqaa - 5 females, 4 males). | Author reflection | Moderately valuable |
| Noubani et al. (39) | Not described | Mental health | Using systems in the community | Participatory GMB workshops | 36 health care providers active in mental health service provision (at least 1 year) from Beirut and Beqaa regions, 15 semi structured interviews conducted with psychologists, nurses, social workers and general practitioners across genders, 21 participants participated in two GMB workshops | Author reflection | Moderately valuable |
| Swierad et al. (40) | Not described | Childhood obesity | Using systems in community and testing the method | CBSD | 16 Chinese American adults. All participants were aged between 20 and 60 years, and 43.8% (7/16) were male. Six participants were born overseas. Participants represented a variety of occupations including nurses, school guidance counsellors, restaurant owners, community health workers, and housewives. | Author reflection | Moderately valuable |
| Trani et al. (41) | Not described | Mental health | Using systems in community and testing the method | A CBSD-informed GMB workshop | Initial sessions - three male and three female community based rehabilitation workers from the Mazar-e-Sharif region and four male CBR workers from Jalalabad. Four participants in the follow-up sessions were from Mazar-e-Sharif, Taloqan, Ghazni and Jalalabad, four regional program offices of the partner NGO. | Author reflection | Moderately valuable |
| Waqa et al. (42) | Not described | Evidence use in food-related policymaking | Using systems in community and testing the method | GMB and a system dynamics approach | 18 participants from the MoHMS (n = 9) and the MOA (n = 9). The majority of participants (72%) were senior managers (such as National Advisors, Directors and Principal level officers) directly involved in policymaking, 28% were middle with potential to share evidence that influences the policymaking process. The majority (72%) were male. | Author reflection | Moderately valuable |
| Zablith et al. (43) | Not described | Non-communicable diseases | Using systems in the community | Semi-structured interviews followed by GMB workshops. | 67 participants. 30 semi-structured interviews: 10 health care providers (physicians, pharmacists, nurses, PHCC managers, 5 male) in the Beqaa, 10 Lebanese (3 men, age range overall 23–60) and 10 Syrian refugee (3 men, age range overall 30–60) community members. All community participants suffered from a chronic condition or self-identified as being at risk of NCD development. First GMB - 10 health care providers (one physician, two pharmacists, six nurses, one PHCC manager); participants had between 3 and 15 years’ experience of working in the Beqaa. Second and third GMB 12 Lebanese community members (41% male, age range 20–50), 15 Syrian refugees (13% male, age range 24–55). All community participants self-identified as having an NCD or a risk factor. | Author reflection | Moderately valuable |
| Zurcher et al. (32) | Food & Fitness (F&F) Initiative | Healthy eating and active living | Using systems in community and testing the method | Five key frameworks and a systems mapping process with community and initiative leaders - Core Theory of Success (CTS), Creative Tension Model (CTM), Hierarchy of Choices (HOC), Levels of Perspective (LoP), Ladder of Inference (LoI), causal loop diagramming | 24 grassroots and institutional members of the six final grantee communities, 10 TA providers, and four WKKF staff participated in structured conversations at the conference. An additional 12 individuals for interviews, ranging from grassroots community members to local evaluators and project directors. | Structured conversations and in-depth phone interviews. | Excellent - very helpful |
